# Microsatellite instability, Epstein–Barr virus, mutation of type II transforming growth factor **β** receptor and BAX in gastric carcinomas in Hong Kong Chinese

**DOI:** 10.1038/sj.bjc.6690092

**Published:** 1999-02

**Authors:** S Y Leung, S T Yuen, L P Chung, K M Chu, M P Wong, F J Branicki, J C I Ho

**Affiliations:** Department of Pathology, The University of Hong Kong, Queen Mary Hospital, Hong Kong, Hong Kong; Department of Surgery, The University of Hong Kong, Queen Mary Hospital, Hong Kong, Hong Kong

**Keywords:** microsatellite instability, gastric carcinoma, Hong Kong Chinese, Epstein–Barr virus, TβRII, BAX

## Abstract

Microsatellite instability (MI), the phenotypic manifestation of mismatch repair failure, is found in a proportion of gastric carcinomas. Little is known of the links between MI and Epstein–Barr virus (EBV) status and clinicopathological elements. Examination of genes mutated through the MI mechanism could also be expected to reveal important information on the carcinogenic pathway. Seventy-nine gastric carcinomas (61 EBV negative, 18 EBV positive) from local Hong Kong Chinese population, an intermediate-incidence area, were examined. Eight microsatellite loci, inclusive of the A10 tract of type II transforming growth factor β receptor (TβR-II), were used to evaluate the MI status. MI in the BAX and insulin-like growth factor II receptor (IGF-IIR) genes were also examined. High-level MI (>40% unstable loci) was detected in ten cases (12.7%) and low-level MI (1–40% unstable loci) in three (3.8%). High-level MI was detected in two EBV-associated cases (11%) and the incidence was similar for the EBV-negative cases (13%). The high-level MIs were significantly associated with intestinal-type tumours (*P* = 0.03) and a more prominent lymphoid infiltrate (*P* = 0.04). Similar associations were noted in the EBV-positive carcinomas. The high-level MIs were more commonly located in the antrum, whereas the EBV-associated carcinomas were mostly located in body. Thirteen cardia cases were negative for both high-level MI and EBV. All patients aged below 55 were MI negative (*P* = 0.049). Of the high-level MIs, 80% had mutation in TβR-II, 40% in BAX and 0% in IGF-IIR. Of low-level MIs, 33% also had TβR-II mutation. These mutations were absent in the MI-negative cases. Of three lymphoepithelioma-like carcinomas, two cases were EBV positive and MI negative, one case was EBV negative but with high-level MI. In conclusion, high-level MIs were present regardless of the EBV status, and were found in a particular clinicopathological subset of gastric carcinoma patient. Inactivation of important growth regulatory genes observed in these carcinomas confirms the importance of MI in carcinogenesis. © 1999 Cancer Research Campaign


*British Journal of Cancer* (1999) **79**(3/4), 582–588
© 1999 Cancer Research Campaign
Article no. bjoc.1998.0092

### Microsatellite instability, Epstein–Barr virus, mutation of type II transforming growth factor β receptor and BAX in gastric carcinomas in Hong Kong Chinese

**SY Leung[1], ST Yuen[1], LP Chung[1], KM Chu[2], MP Wong[1], FJ Branicki[2] and JCI Ho[1]**

[1]Department of Pathology and [2]Department of Surgery, The University of Hong Kong, Queen Mary Hospital, Hong Kong

**Summary** Microsatellite instability (MI), the phenotypic manifestation of mismatch repair failure, is found in a proportion of gastric carcinomas. Little is known of the links between MI and Epstein–Barr virus (EBV) status and clinicopathological elements. Examination of genes mutated through the MI mechanism could also be expected to reveal important information on the carcinogenic pathway. Seventy-nine gastric carcinomas (61 EBV negative, 18 EBV positive) from local Hong Kong Chinese population, an intermediate-incidence area, were examined. Eight microsatellite loci, inclusive of the A10 tract of type II transforming growth factor β receptor (TβR-II), were used to evaluate the MI status. MI in the BAX and insulin-like growth factor II receptor (IGF-IIR) genes were also examined. High-level MI (>40% unstable loci) was detected in ten cases (12.7%) and low-level MI (1–40% unstable loci) in three (3.8%). High-level MI was detected in two EBV-associated cases (11%) and the incidence was similar for the EBV-negative cases (13%). The high-level MIs were significantly associated with intestinal-type tumours ($P = 0.03$) and a more prominent lymphoid infiltrate ($P = 0.04$). Similar associations were noted in the EBV-positive carcinomas. The high-level MIs were more commonly located in the antrum, whereas the EBV-associated carcinomas were mostly located in body. Thirteen cardia cases were negative for both high-level MI and EBV. All patients aged below 55 were MI negative ($P = 0.049$). Of the high-level MIs, 80% had mutation in TβR-II, 40% in BAX and 0% in IGF-IIR. Of low-level MIs, 33% also had TβR-II mutation. These mutations were absent in the MI-negative cases. Of three lymphoepithelioma-like carcinomas, two cases were EBV positive and MI negative, one case was EBV negative but with high-level MI. In conclusion, high-level MIs were present regardless of the EBV status, and were found in a particular clinicopathological subset of gastric carcinoma patient. Inactivation of important growth regulatory genes observed in these carcinomas confirms the importance of MI in carcinogenesis.

**Keywords:** microsatellite instability; gastric carcinoma; Hong Kong Chinese; Epstein–Barr virus; TβRII; BAX

Microsatellite instability (MI), the phenotypic manifestation of mismatch repair failure, is detectable in a wide variety of human cancers. It is particularly seen in patients with the hereditary non-polyposis colon cancer (HNPCC) syndrome, in which the affected individual is at risk of developing carcinoma both at an early age and at a range of sites such as the colon, endometrium, urinary tract and stomach (Aaltonen et al, 1993; Marra and Boland, 1995; Lynch et al, 1996). Germline mutation in one of the DNA mismatch repair genes is responsible for a significant proportion of individuals affected by this syndrome (Fishel et al, 1993; Bronner et al, 1994; Liu et al, 1994; Nicolaides et al, 1994; Papadopoulos et al, 1994). MI has also been detected in a variable proportion of the sporadic form of cancer arising in these organs (Eshleman and Markowitz, 1995).

Although gastric cancer, worldwide, is deemed to be both the second most common malignancy and second leading cause of cancer death (Parkin et al, 1993; Pisani et al, 1993), the incidence varies markedly in different populations. The incidence of molecular genetic changes is known to vary; for example, the series of reports from high incidence areas such as Japan, Italy, Portugal and Korea show a range for MI varying from 15% to 39% (Han et al, 1993; Chong et al, 1994; Rhyu et al, 1994; Strickler et al, 1994; Lin et al, 1995; Chung et al, 1996; dos Santos et al, 1996; Ohue et al, 1996; Renault et al, 1996). Although MI was noted in a high percentage of gastric cancers with a positive family history (Akiyama et al, 1996; Keller et al, 1996; Ottini et al, 1997), germline or somatic mutations of the mismatch repetition and repair (MMR) genes were rarely reported (Akiyama et al, 1996; Keller et al, 1996). Thus, the mechanism leading to the mismatch repair failure in gastric carcinomas remains largely unknown.

The association between Epstein–Barr virus (EBV) and gastric cancer also varies among different populations, with the highest positive association occurring in Caucasian populations (Shibata and Weiss, 1992; Ott et al, 1994) and a low association seen in the Japanese (Tokunaga et al, 1993). EBV is found in more than 80% of a specific histological variant of gastric carcinoma, variably termed lymphoepithelioma-like carcinoma (LELC), medullary or gastric carcinoma with lymphoid stroma (Nakamura et al, 1994). This variant is characterized by the presence of a poorly differentiated morphology, a circumscribed border, an intense lymphoid reaction and a better prognosis (Watanabe et al, 1976). The possible relationship between EBV, MI and LELC is largely unexplored except in one study from Japan, in which all eight EBV-associated gastric cancers were negative for MI (Chong et al, 1994).

Previous data have shown that the mutator phenotype conferred by MI has a significant role in carcinogenesis, in which it targets

*Received 27 January 1998*
*Revised 1 April 1998*
*Accepted 14 May 1998*

*Correspondence to:* Suet Yi Leung, Department of Pathology, Queen Mary Hospital, Hong Kong specific genes that play an important role in the growth-regulatory pathway. The polyadenine tract in the type II transforming growth factor β receptor (TβR-II) gene is one of the most commonly mutated genes in MI-positive cancers. This leads to the inactivation of an important growth-suppressive pathway (Markowitz et al, 1995; Myeroff et al, 1995; Parsons et al, 1995). Other genes harbouring polynucleotide tracts are being increasingly demonstrated to be affected, revealing the importance of MI in the carcinogenic steps.

In Hong Kong, the population is more than 90% southern Chinese and the annual incidence of gastric cancer is 13.2, an intermediate incidence area (Hong Kong Cancer Registry, 1996). This study examines the incidence of MI in gastric cancer in Hong Kong Chinese when correlated with various clinicopathological parameters. The relationship of MI with EBV, mutation of the polynucleotide tract of TβR-II, BAX and insulin-like growth factor II receptor (IGF-IIR) were particularly examined, in an attempt to further elucidate the role of MI in carcinogenesis.

### MATERIALS AND METHODS

### Materials

Seventy-nine cases of gastric carcinoma from Hong Kong Chinese were studied, with the series consisting of 18 cases of EBV-associated and 61 EBV-negative gastric carcinomas. The EBV-associated cases were derived from the screening of 290 non-consecutive gastric carcinomas from 1986 to 1997 using in situ hybridization to detect EBV-encoded RNAs (EBER), as described previously (Yuen et al, 1994). The EBV-negative cases were from gastrectomy specimens performed in the period 1993–97 for whom frozen blocks were still available. Paired tumour and normal tissue blocks were selected. The normal tissue was dissected away as much as possible in the tumour blocks, and cryostat or paraffin sections were cut and examined to confirm the tumour cell percentage. Only those tumour blocks containing more than 30% tumour cells were selected. The normal blocks were similarly examined to ensure the absence of tumour cell contamination. Sections were cut, deparaffinized as needed and suspended in proteinase K buffer. DNA was purified using a standard proteinase K digestion, phenol–chloroform extraction method.

### Methods

Eight loci were used to evaluate the MI status. These included five dinucleotide repeats (Tp53, D18S58, D18S57, D2S123, D5S346) and three mononucleotide repeats of polyadenine tracts (BAT26, BAT40 and BAT-RII). Tp53, D18S58, D18S57, D2S123 and D5S346 were purchased from Research Genetics (Huntsville, AL, USA). BAT26 and BAT40 were synthesized according to the sequence published previously (Liu et al, 1996). For D2S123 and D18S58, additional sets of primers synthesized according to the published sequence (Liu et al, 1996) were also used. The BAT-RII, synthesized according to Myeroff et al (1995), amplify a 73-bp region of the TβR-II gene. These span nucleotides 665–737, which contain a polyadenine tract. In 87% of cases, seven to eight loci were analysed. In the remaining 13%, at least five of these eight loci were analysed, including both dinucleotide and polyadenine repeats.

The primer sequences for BAX were the same as those described by Rampino et al (1997), and amplified a segment containing eight consecutive deoxyguanosines spanning codons 38–41. The primer for IGF-IIR amplified a segment spanning nucleotides 4030–4140, which contained an eight-deoxyguanine repeat (Souza et al, 1996). All cases with microsatellite instability were analysed for frameshift mutation in the BAX and IGF-IIR gene. An additional 48 MI-negative cases were also analysed for BAX mutation.

The polymerase chain reaction (PCR) was performed in a 10-µl reaction solution containing 50 ng of DNA, 10 mM Tris (pH 8.3), 50 mM potassium chloride, 2–3 mM $Mg^{2+}$, 200 µM dNTP, 1 µCi of [α-$^{32}$P]dCTP, 0.2–1 µM of each primers and 0.1 U *Taq* polymerase. Hot-start reaction was performed by preheating the mixture in the thermocycler at 95°C for 5 min, then 80°C before adding the *Taq* polymerase. An initial denaturation step of 95°C for 5 min and 25–40 cycles including 95°C for 45 s (1 min), 1 min (1.5 min) in 52–64°C annealing temperature according to the specific primers, and 72°C for 1 min (2 min) in frozen DNA (paraffin DNA) was performed, followed by a final extension of 5 min at 72°C.

The PCR products were diluted by loading buffer, heated at 95°C for 5 min and loaded onto 7% vertical polyacrylamide gel. After electrophoresis, the gels were fixed and dried and exposed to radiographic film for between 12 h and 7 days. The band patterns between the tumour and non-tumour tissue were compared and scored by two independent investigators. All cases with alteration of band sizes in the tumour tissue compared with normal were repeated once. The number of loci showing microsatellite instability were recorded in each case. Cases with >40% unstable loci, including both types of repeats, were classified as high-level MIs. Cases with 1–40% unstable loci were classified as low-level MIs.

### RESULTS

Microsatellite instability was detected in 13 cases (16.5%); including ten (12.7%) high-level MIs and three (3.8%) low-level MIs (Table 1). The high-level MIs tended to have lots of unstable loci involving both dinucleotide and mononucleotide repeats, whereas the low-level MIs tended to affect the mononucleotide repeats only. Although two of the high-level MIs were also EBV associated, the incidence of high-level MI was similar for the EBV-positive (11%) and EBV-negative cases (13%). Of the ten high-level MIs, eight (80%) had frameshift mutation in the A10 tract of TβR-II and four (40%) had frameshift mutation in the G8 tract of BAX gene. All cases with BAX mutation also had mutation of the TβR-II gene. One of the three (33%) low-level MIs had mutation of the A10 tract of TβR-II. No mutation of the IGF-IIR gene was detected in any of the MI cases. Mutation of TβR-II and BAX was not detected in any of the MI-negative cases. Representative results of the microsatellite analysis are shown in Figures 1 and 2.

The relationship between MI and EBV status and clinicopathological parameters is shown in Tables 2 and 3. All the high-level MIs were of intestinal type, which differs significantly from the low-level and negative MIs (Fisher exact test, $P = 0.03$). The high-level MIs also had a significantly more prominent lymphoid infiltrate (Fisher exact test, $P = 0.04$). Three cases had the morphological characteristic of LELC, two were EBV positive and MI negative, whereas one was EBV negative but with high-level MI. The EBV-associated cases were most commonly located in the gastric body (chi-squared test, $P < 0.00001$), whereas the high-level MIs were mostly located in the antrum but did not reach

© Cancer Research Campaign 1999

**Table 1** Clinicopathological features, mutation of the TβR-II, BAX and IGF-IIR genes in microsatellite instability (MI)-positive gastric carcinomas

| Patient | 1 | 2 | 3 | 4 | 5 | 6 | 7 | 8 | 9 | 10 | 11 | 12 | 13 |
|---|---|---|---|---|---|---|---|---|---|---|---|---|---|
| Sex | F | M | F | M | F | M | M | M | F | M | F | F | M |
| Age | 77 | 59 | 77 | 78 | 65 | 66 | 63 | 57 | 79 | 79 | 79 | 73 | 55 |
| Tumour site | C | C | A | B | B | A | A | A | A | B | A | A | A |
| Laurens' type | I | I | D | I | I | I | I | I | I | I[b] | I | I | I |
| Lymphoid infiltrate | 1 | 0 | 0 | 2 | 2 | 0 | 2 | 2 | 1 | 3 | 3 | 0 | 2 |
| EBV status | 0 | 0 | 0 | 1 | 1 | 0 | 0 | 0 | 0 | 0 | 0 | 0 | 0 |
| Tp53 | 0 | 0 | 0 | 0 | 1 | 1 | 1 | 1 | 1 | 1 | 1 | 1 | 1 |
| D18S58 | 0 | 0 | 0 | ND | 1 | 1 | 1 | 1 | 1 | 0 | 1 | 1 | 1 |
| D18S57 | 0 | ND | 0 | ND | ND | 1 | 0 | 0 | 0 | ND | ND | ND | 1 |
| D2S123 | 0 | 0 | 0 | 1 | 1 | 0 | 0 | 1 | 0 | 1 | 1 | 1 | 1 |
| D5S346 | 0 | 0 | 0 | 0 | 0 | 1 | 1 | 0 | 1 | 1 | ND | 1 | 1 |
| BAT26 | 0 | 0 | 0 | 0 | 0 | 0 | 1 | 1 | 1 | 1 | 1 | 1 | 1 |
| BAT40 | 1 | 1 | 1 | 1 | 1 | 1 | 1 | 1 | 1 | 1 | 1 | 1 | 1 |
| BAT-RII | 0 | 0 | 1 | 1 | 0 | 0 | 1 | 1 | 1 | 1 | 1 | 1 | 1 |
| No. of MI/loci[a] | 1/8 | 1/7 | 2/8 | 3/6 | 4/7 | 5/8 | 6/8 | 6/8 | 6/8 | 6/7 | 6/6 | 7/7 | 8/8 |
| % Unstable loci | 13 | 14 | 25 | 50 | 57 | 63 | 75 | 75 | 75 | 86 | 100 | 100 | 100 |
| BAX (G8 tract) | 0 | 0 | 0 | 1 | 0 | 0 | 0 | 1 | 1 | 0 | 0 | 1 | 0 |
| IGF-IIR (G8 tract) | 0 | 0 | 0 | 0 | 0 | 0 | 0 | 0 | 0 | 0 | 0 | 0 | 0 |

Tumour site: C, cardia; B, body; A, antrum; Laurens' tumour type: I, intestinal; D, diffuse; Lymphoid infiltrate: 0, minimal; 1, mild; 2, moderate; 3, intense; EBV status; 0, negative; 1, positive; microsatellite instability; 0, negative; 1, positive; ND, not determined; [a]inclusive of BAT-RII (A10 tract of TβRII); [b]this is a case of lymphoepithelioma-like carcinoma.

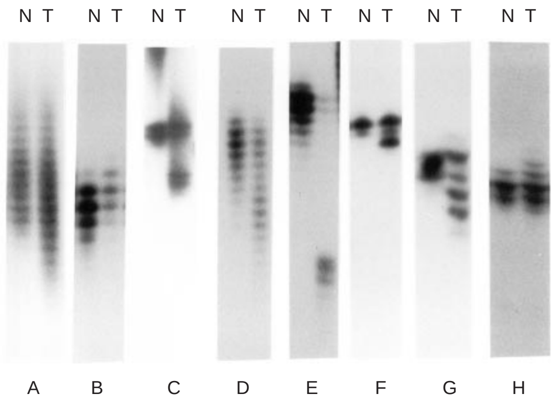

**Figure 1** Microsatellite alteration at various loci in gastric carcinoma. (**A**) Tp53; (**B**) D2S123; (**C**) D18S58; (**D**) BAT40; (**E**) BAT26; (**F**) BAT-RII; (**G**) D18S57; (**H**) D5S346. N, normal tissue; T, tumour tissue

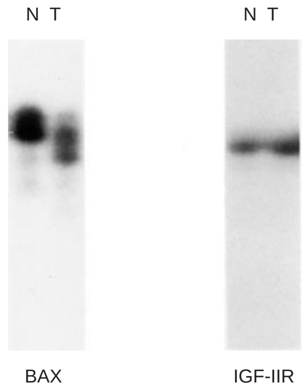

**Figure 2** Frameshift mutation is noted in the BAX gene but absent in the IGF-IIR gene. N, normal tissue; T, tumour tissue statistical significance. There were 13 cases from the cardia and all were negative for high-level MI and EBV. There was no significant association for high-level MIs or EBV status with sex, tumour differentiation, tumour depth of invasion or lymph node metastasis.

Both high-level and low-level MIs were found in patients aged 55 or above. None of the 15 patients aged below 55 showed MI. Although comparison of high-level MIs compared with low/negative MIs did not quite reach statistical significance (Fisher exact test, $P = 0.1$), the difference between negative and low-/high-level MIs was significant (chi-squared test, $P = 0.049$).

### DISCUSSION

This study detected the presence of high-level MI in 12.7% of gastric carcinomas in Hong Kong Chinese. Though the overall incidence of MI in our series is low (16.5%), the incidence of high-level MI is either similar (Han et al, 1993; Chong et al, 1994; Strickler et al, 1994; Ohue et al, 1996; Ottini et al, 1997) or only slightly lower (Rhyu et al, 1994; Chung et al, 1996; dos Santos et al, 1996) when compared with many large series from high-incidence areas. Although the low-level MI cases may simply represent an inherent instability of repeat sequences in cancer tissue, the high-level MI cases are highly suggestive of defects in the DNA mismatch repair systems. In fact, a criteria of more than 40% unstable loci were advocated by some groups to qualify a case as MI (Bocker et al, 1997; Dietmaier et al, 1997). Our study has identified a specific clinicopathological profile in patients with high-level MIs: they are all elderly patients, the tumours are of intestinal type and show a significantly more intense lymphoid infiltrate. Also, location in the antrum is more common.

Our results confirm the previous observations of dos Santos et al (1996), who demonstrated more prominent lymphoid infiltrate in gastric carcinoma when there was a high level of MI. This invites comparison with those MI-positive colorectal carcinomas in which Crohn's disease-like lymphoid reaction is also more commonly seen (Kim et al, 1994). Our study included three LELC cases; two were EBV associated with negative MI, and one was EBV

 © Cancer Research Campaign 1999

**Table 2** Microsatellite instability and clinicopathological features in 79 gastric carcinomas

| | **MI-negative (0% locus)** | **Low-level MI (1–40% loci)** | **High-level MI (>40% loci)** | **Total** | ***P*-value[a]** |
|---|---|---|---|---|---|
| Sex | | | | | |
| M | 49 (14) | 1 | 6 (1) | 56 | |
| F | 17 (2) | 2 | 4 (1) | 23 | |
| Age groups (years) | | | | | |
| <45 | 12 (4) | 0 | 0 | 12 | *P* = 0.1[b] |
| 45–54 | 3 | 0 | 0 | 3 | High vs low/negative MI, |
| 55–64 | 20 (6) | 1 | 3 | 24 | <55 vs 55+ |
| 65+ | 31 (6) | 2 | 7 (2) | 40 | |
| Differentiation | | | | | |
| Moderate | 26 (5) | 2 | 5 | 33 | |
| Poor | 40 (11) | 1 | 5 (2) | 46 | |
| Tumour type | | | | | |
| Intestinal | 45 (14[c]) | 2 | 10[d] (2) | 57 | *P* = 0.03 |
| Diffuse | 13 | 1 | 0 | 14 | High vs low/negative MI, |
| Mixed | 8 (2) | 0 | 0 | 8 | Intestinal vs diffuse/mixed |
| Tumour site | | | | | |
| Cardia | 11 | 2 | 0 | 13 | |
| Body | 21 (12) | 0 | 3 (2) | 24 | |
| Antrum | 34 (4) | 1 | 7 | 42 | |
| Lymphoid infiltrate | | | | | |
| Minimal/mild | 42 (5) | 3 | 3 | 48 | *P* = 0.04 |
| Moderate/severe | 24 (11) | 0 | 7 (2) | 31 | High vs low/negative MI |
| Tumour level | | | | | |
| Submucosa | 2 (1) | 0 | 0 | 2 | |
| Muscle | 7 (1) | 1 | 4 | 12 | |
| Serosa | 57 (14) | 2 | 6 (2) | 65 | |
| Lymph node | | | | | |
| Negative | 13 (2) | 0 | 2 | 15 | |
| Positive | 53 (14) | 3 | 8 (2) | 64 | |

[a]Fisher exact test, one tail; [b]MI negative vs low-/high-level MI, <55 vs 55+, *P* = 0.049; numbers in parentheses equal to number of EBV-positive cases; [c]inclusive of two cases of LELC; [d]inclusive of one case of LELC.

negative but showed high-level MI. There is also a reported series of 13 colonic medullary carcinomas which were all EBV negative but MI positive (Ruschoff et al, 1997). Thus, it appears that MI may contribute to a subset of carcinoma with the morphological features of LELC/medullary carcinoma. The lymphoid reaction in LELC with MI may be triggered by more frequent genetic errors resulting in formation of mutated immunogenic proteins, or, in the EBV-associated cases, by expression of certain viral antigens in the carcinoma cells.

The association of high-level MI with intestinal type tumour and antral location correlate well with the results of Chung et al (1996) and Ottini et al (1997). Although EBV-associated cases were also more commonly seen in intestinal type tumours with intense lymphoid infiltrate, these were mostly located in the gastric body. In this series, none of the carcinoma in the cardia were associated with EBV or high-level MI. This poses an interesting question concerning regional susceptibility of the stomach to different carcinogenic pathways.

The mutator phenotype conferred by MI plays an important role in carcinogenesis. In previous series, insertion/deletions in the polyadenine tract of TGF-βRII genes has been detected in more than 70% of gastric carcinomas with MI (Myeroff et al, 1995; Chung et al, 1996; Yamamoto et al, 1997). We also found mutations of TβR-II in 80% of gastric carcinomas with high-level MI, one of which was seen in an EBV-associated case. It is interesting to note that in 70% of the tumours with high-level MIs, there is a moderate to intense lymphoid infiltrate. Now, as TGF-β is a potent growth inhibitor of epithelial cells as well as a potent immunosuppressive cytokine (Kehrl, 1991; Wojtowicz Praga, 1997), the cancer cells with mutated TβR-II may be both released from the growth-inhibitory effect of TGF-β and yet benefit from its immunosuppressive effect.

The BAX gene is an antagonist of Bcl-2, and promotes apoptosis. Inactivation of this gene due to frameshift mutation in the GI tract was first reported to occur in more than 50% of MI-positive colorectal carcinomas (Rampino et al, 1997). Two subsequent series reported a total of 20 BAX frameshift mutations in 31 MI-positive gastric carcinomas (65%) (Chung et al, 1997; Yamamoto et al, 1997). In the current series, we found BAX mutation in 40% of the high-level MIs. None of the low-level MIs or MI-negative cases showed this type of frameshift mutation. Moreover, all cases with BAX mutation also showed mutation in TβR-II. This supports the notion that, although MI causes mutation in a random fashion, only specific genes involved in control of cell cycle or apoptosis were selected for in the carcinogenic process. Interestingly, none of the MI-positive cases in our series showed IGF-IIR mutation. There have only been a few reports on mutation of this gene in gastric cancers but in most series the incidence of mutation is low, ranging from 8% to 33% (Souza et al, 1996; Chung et al, 1997; Ouyang et al, 1997; Yamamoto et al, 1997).

*© Cancer Research Campaign 1999*

**Table 3** EBV and the clinicopathological features in 79 gastric carcinomas

| | EBV-negative | EBV positive | Total | *P*-value[a] |
|---|---|---|---|---|
| Sex | | | | |
| M | 41 (5) | 15 (1) | 56 | |
| F | 20 (3) | 3 (1) | 23 | |
| Age groups (years) | | | | |
| <45 | 8 | 4 | 12 | |
| 45–54 | 3 | 0 | 3 | |
| 55–64 | 18 (3) | 6 | 24 | |
| 65+ | 32 (5) | 8 (2) | 40 | |
| Differentiation | | | | |
| Moderate | 28 (5) | 5 | 33 | |
| Poor | 33 (3) | 13 (2) | 46 | |
| Tumour type | | | | |
| Intestinal | 41 (8[b]) | 16[c] (2) | 57 | $P = 0.07$ |
| Diffuse | 14 | 0 | 14 | Intestinal vs diffuse/mixed |
| Mixed | 6 | 2 | 8 | |
| Tumour site | | | | |
| Cardia | 13 | 0 | 13 | $P < 0.00001$ |
| Body | 10 (1) | 14 (2) | 24 | |
| Antrum | 38 (7) | 4 | 42 | |
| Lymphoid infiltrate | | | | |
| Minimal/mild | 43 (3) | 5 | 48 | $P = 0.001$ |
| Moderate/severe | 18 (5) | 13 (2) | 31 | |
| Tumour level | | | | |
| Submucosa | 1 | 1 | 2 | |
| Muscle | 11 (4) | 1 | 12 | |
| Serosa | 49 (4) | 16 (2) | 65 | |
| Lymph node | | | | |
| Negative | 13 (2) | 2 | 15 | |
| Positive | 48 (6) | 16 (2) | 64 | |

[a]Chi-squared test; numbers in parentheses equal to number of high-level MI cases; [b]inclusive of one case of LELC; [c]inclusive of two cases of LELC.

Also, tumours tend to be positive for either TβR-II or IGF-IIR mutation, but not both (Souza et al, 1996). This can be explained by the fact that IGF-IIR is required for the activation of the TGF-β1 complex from its latent form so that they may constitute serial points in the same carcinogenic pathway (Souza et al, 1996). Because most of the high-level MIs are positive for TβR-II mutation in our series, the absence of IGF-IIR mutation is consistent with the hypothesis. In summary, inactivation of TβR-II appears to be the most common and important pathway targeted by MI in gastric carcinomas, followed by BAX gene, whereas mutation of IGF-IIR is relatively uncommon.

We found high-level MI in both EBV-negative as well as EBV-positive gastric carcinomas. This is in contrast to a previous study by Chong et al (1994), who found no MI in eight EBV-associated gastric carcinomas. Whether MI is present in other EBV-associated carcinomas is not known. The possibility that MI is caused by the human immunodeficiency virus (HIV) has been raised by a group of investigators who detected a high incidence of MI in B-cell non-Hodgkin's lymphoma and in Kaposi sarcoma developing in patients with the acquired immunodeficiency syndrome (AIDS) (Bedi et al, 1995). Because a high proportion of B-cell non-Hodgkin's lymphoma in AIDS patients are associated with EBV (Shibata et al, 1993; Hamilton Dutoit and Pallesen, 1994; Anagnostopoulos and Hummel, 1996), and human herpesvirus 8 is present in Kaposi sarcoma (Schalling et al, 1995), it would be most useful to determine whether the MI observed in these tumours was related to the HIV or the other two herpes viruses.

Although there is a high incidence of MI in young colorectal cancer patients both in Hong Kong (Chan et al, 1997) and elsewhere (Liu et al, 1995), this does not seem to be so in gastric cancer. None of the 12 gastric cancer patients aged below 45 in our series showed MI, and all the MI cases were aged 55 or above. Thus, although gastric cancer is within the spectrum of HNPCC-related tumours, it cannot account for the young gastric cancer in our population.

In summary, MI may constitute an important carcinogenic pathway in a specific clinicopathological subgroup of gastric carcinoma through inactivation of specific growth regulatory genes. The mechanism leading to MI in these sporadic gastric carcinomas is an area for further studies.

### ACKNOWLEDGEMENTS

We thank Miss Annie SY Chan and Mr Samson WC Shum for the excellent technical assistance, Dr Robert J Collins for editing the manuscript. Part of this work constitutes the M.D. thesis of Dr SY Leung. This work is supported by the Michael and Betty Kadoorie Foundation Cancer Research Genetics Fund.

 © Cancer Research Campaign 1999

### REFERENCES

Aaltonen LA, Peltomaki P, Leach FS, Sistonen P, Pylkkanen L, Mecklin JP, Jarvinen H, Powell SM, Jen J, Hamilton SR, Petersen GM, Kinzler KW, Vogelstein B and De La Chapelle A (1993) Clues to the pathogenesis of familial colorectal cancer. *Science* **260**: 812–816

Akiyama Y, Nakasaki H, Nihei Z, Iwama T, Nomizu T, Utsunomiya J and Yuasa Y (1996) Frequent microsatellite instabilities and analyses of the related genes in familial gastric cancers. *Jpn J Cancer Res* **87**: 595–601

Anagnostopoulos I and Hummel M (1996) Epstein–Barr virus in tumours. *Histopathology* **29**: 297–315

Bedi GC, Westra WH, Farzadegan H, Pitha PM and Sidransky D (1995) Microsatellite instability in primary neoplasms from HIV + patients. *Nature Med* **1**: 65–68

Bocker T, Diermann J, Friedl W, Gebert J, Holinski Feder E, Karner H, Von Knebel Doeberitz M, Koelble K, Moeslein G, Schackert HK, Wirtz HC, Fishel R and Ruschoff J (1997) Microsatellite instability analysis: a multicenter study for reliability and quality control. *Cancer Res* **57**: 4739–4743

Bronner CE, Baker SM, Morrison PT, Warren G, Smith LG, Lescoe MK, Kane M, Earabino C, Lipford J, Lindblom A, Tannergard P, Bollag RJ, Godwin AR, Ward DC, Nordenskjold M, Fishel R, Kolodner R and Liskay RM (1994) Mutation in the DNA mismatch repair gene homologue hMLH1 is associated with hereditary non-polyposis colon cancer. *Nature* **368**: 258–261

Chan TL, Yuen ST, Chung LP, Ho J, Leung SY, Chan AS and Chan LC (1997) Replication errors (RER) and mismatch repair (MMR) gene mutations in young colorectal carcinoma patients in Hong Kong Chinese. In *Proceedings of Keystone Symposium on Molecular and Cellular Biology – Genetics of Human Cancer: Pathogenesis and Diagnosis, Colorado*, p. 9

Chong JM, Fukayama M, Hayashi Y, Takizawa T, Koike M, Konishi M, Kikuchi Yanoshita R and Miyaki M (1994) Microsatellite instability in the progression of gastric carcinoma. *Cancer Res* **54**: 4595–4597

Chung YJ, Song JM, Lee JY, Jung YT, Seo EJ, Choi SW and Rhyu MG (1996) Microsatellite instability-associated mutations associate preferentially with the intestinal type of primary gastric carcinomas in a high-risk population. *Cancer Res* **56**: 4662–4665

Chung YJ, Park SW, Song JM, Lee KY, Seo EJ, Choi SW and Rhyu MG (1997) Evidence of genetic progression in human gastric carcinomas with microsatellite instability. *Oncogene* **15**: 1719–1726

Dietmaier W, Wallinger S, Bocker T, Kullmann F, Fishel R and Ruschoff J (1997) Diagnostic microsatellite instability: definition and correlation with mismatch repair protein expression. *Cancer Res* **57**: 4749–4756

Dos Santos NR, Seruca R, Constancia M, Seixas M and Sobrinho Simoes M (1996) Microsatellite instability at multiple loci in gastric carcinoma: clinicopathologic implications and prognosis. *Gastroenterology* **110**: 38–44

Eshleman JR and Markowitz SD (1995) Microsatellite instability in inherited and sporadic neoplasms. *Curr Opinion Oncol* **7**: 83–89

Fishel R, Lescoe MK, Rao MR, Copeland NG, Jenkins NA, Garber J, Kane M and Kolodner R (1993) The human mutator gene homolog MSH2 and its association with hereditary nonpolyposis colon cancer. *Cell* **75**: 1027–1038

Hamilton Dutoit SJ and Pallesen G (1994) Detection of Epstein–Barr virus small RNAs in routine paraffin sections using non-isotopic RNA/RNA in situ hybridization. *Histopathology* **25**: 101–111

Han HJ, Yanagisawa A, Kato Y, Park JG and Nakamura Y (1993) Genetic instability in pancreatic cancer and poorly differentiated type of gastric cancer. *Cancer Res* **53**: 5087–5089

Hong Kong Cancer Registry (1996) *Cancer Incidence and Mortality in Hong Kong 1992*, p. 4. Hospital Authority: Hong Kong

Kehrl JH (1991) Transforming growth factor-beta: an important mediator of immunoregulation. *Int J Cell Cloning* **9**: 438–450

Keller G, Grimm V, Vogelsang H, Bischoff P, Mueller J, Siewert JR and Hofler H (1996) Analysis for microsatellite instability and mutations of the DNA mismatch repair gene hMLH1 in familial gastric cancer. *Int J Cancer* **68**: 571–576

Kim H, Jen J, Vogelstein B and Hamilton SR (1994) Clinical and pathological characteristics of sporadic colorectal carcinomas with DNA replication errors in microsatellite sequences. *Am J Pathol* **145**: 148–156

Lin JT, Wu MS, Shun CT, Lee WJ, Wang JT, Wang TH and Sheu JC (1995) Microsatellite instability in gastric carcinoma with special references to histopathology and cancer stages. *Eur J Cancer* **31A**: 1879–1882

Liu B, Parsons RE, Hamilton SR, Petersen GM, Lynch HT, Watson P, Markowitz S, Willson JK, Green J, De La Chapelle A, Kinzler KW and Vogelstein B (1994) hMSH2 mutations in hereditary nonpolyposis colorectal cancer kindreds. *Cancer Res* **54**: 4590–4594

Liu B, Farrington SM, Petersen GM, Hamilton SR, Parsons R, Papadopoulos N, Fujiwara T, Jen J, Kinzler KW, Wyllie AH, Vogelstein B and Dunlop MG (1995) Genetic instability occurs in the majority of young patients with colorectal cancer. *Nature Med* **1**: 348–352

Liu B, Parsons R, Papadopoulos N, Nicolaides NC, Lynch HT, Watson P, Jass JR, Dunlop M, Wyllie A, Peltomaki P, De La Chapelle A, Hamilton SR, Vogelstein B and Kinzler KW (1996) Analysis of mismatch repair genes in hereditary non-polyposis colorectal cancer patients. *Nature Med* **2**: 169–174

Lynch HT, Smyrk T and Lynch JF (1996) Overview of natural history, pathology, molecular genetics and management of HNPCC (Lynch syndrome). *Int J Cancer* **69**: 38–43

Markowitz S, Wang J, Myeroff L, Parsons R, Sun L, Lutterbaugh J, Fan RS, Zborowska E, Kinzler KW, Vogelstein B, Brattain M and Willson JK (1995) Inactivation of the type II TGF-beta receptor in colon cancer cells with microsatellite instability. *Science* **268**: 1336–1338

Marra G and Boland CR (1995) Hereditary nonpolyposis colorectal cancer: the syndrome, the genes, and historical perspectives. *J Natl Cancer Inst* **87**: 1114–1125

Myeroff LL, Parsons R, Kim SJ, Hedrick L, Cho KR, Orth K, Mathis M, Kinzler KW, Lutterbaugh J, Park K, Bang YJ, Lee HY, Park JG, Lynch HT, Roberts AB, Vogelstein B and Markowitz SD (1995) A transforming growth factor beta receptor type II gene mutation common in colon and gastric but rare in endometrial cancers with microsatellite instability. *Cancer Res* **55**: 5545–5547

Nakamura S, Ueki T, Yao T, Ueyama T and Tsuneyoshi M (1994) Epstein–Barr virus in gastric carcinoma with lymphoid stroma. Special reference to its detection by the polymerase chain reaction and in situ hybridization in 99 tumors, including a morphologic analysis. *Cancer* **73**: 2239–2249

Nicolaides NC, Papadopoulos N, Liu B, Wei YF, Carter KC, Ruben SM, Rosen CA, Haseltine WA, Fleischmann RD, Fraser CM, Adams MD, Venter JC, Dunlop MG, Hamilton SR, Petersen GM, De La Chapelle A, Vogelstein B and Kinzler KW (1994) Mutations of two PMS homologues in hereditary nonpolyposis colon cancer. *Nature* **371**: 75–80

Ohue M, Tomita N, Monden T, Miyoshi Y, Ohnishi T, Izawa H, Kawabata Y, Sasaki M, Sekimoto M, Nishisho I, Shiozaki H and Monden M (1996) Mutations of the transforming growth factor beta type II receptor gene and microsatellite instability in gastric cancer. *Int J Cancer* **68**: 203–206

Ott G, Kirchner T and Muller Hermelink HK (1994) Monoclonal Epstein–Barr virus genomes but lack of EBV-related protein expression in different types of gastric carcinoma. *Histopathology* **25**: 323–329

Ottini L, Palli D, Falchetti M, D'Amico C, Amorosi A, Saieva C, Calzolari A, Cimoli F, Tatarelli C, Marchis LD, Masala G, Mariani-Costantini R and Cama A (1997) Microsatellite instability in gastric cancer is associated with tumor location and family history in a high-risk population from Tuscany. *Cancer Res* **57**: 4523–4529

Ouyang H, Shiwaku HO, Hagiwara H, Miura K, Abe T, Kato Y, Ohtani H, Shiba K, Souza RF, Meltzer SJ and Horii A (1997) The insulin-like growth factor II receptor gene is mutated in genetically unstable cancers of the endometrium, stomach, and colorectum. *Cancer Res* **57**: 1851–1854

Papadopoulos N, Nicolaides NC, Wei YF, Ruben SM, Carter KC, Rosen C, Haseltine WA, Fleischmann RD, Fraser CM, Adams MD, Venter JC, Hamilton SR, Petersen GM, Watson P, Lynch HT, Peltomaki P, Mecklin JP, De La Chapelle A, Kinzler KW and Vogelstein B (1994) Mutation of a mutL homolog in hereditary colon cancer. *Science* **263**: 1625–1629

Parkin DM, Pisani P and Ferlay J (1993) Estimates of the worldwide incidence of eighteen major cancers in 1985. *Int J Cancer* **54**: 594–606

Parsons R, Myeroff LL, Liu B, Willson JK, Markowitz SD, Kinzler KW and Vogelstein B (1995) Microsatellite instability and mutations of the transforming growth factor beta type II receptor gene in colorectal cancer. *Cancer Res* **55**: 5548–5550

Pisani P, Parkin DM and Ferlay J (1993) Estimates of the worldwide mortality from eighteen major cancers in 1985. Implications for prevention and projections of future burden. *Int J Cancer* **55**: 891–903

Rampino N, Yamamoto H, Ionov Y, Li Y, Sawai H, Reed JC and Perucho M (1997) Somatic frameshift mutations in the BAX gene in colon cancers of the microsatellite mutator phenotype. *Science* **275**: 967–969

Renault B, Calistri D, Buonsanti G, Nanni O, Amadori D and Ranzani GN (1996) Microsatellite instability and mutations of p53 and TGF-beta RII genes in gastric cancer. *Hum Genet* **98**: 601–607

Rhyu MG, Park WS and Meltzer SJ (1994) Microsatellite instability occurs frequently in human gastric carcinoma. *Oncogene* **9**: 29–32

Ruschoff J, Dietmaier W, Luttges J, Seitz G, Bocker T, Zirngibl H, Schlegel J, Schackert HK, Jauch KW and Hofstaedter F (1997) Poorly differentiated colonic adenocarcinoma, medullary type: clinical, phenotypic, and molecular characteristics. *Am J Pathol* **150**: 1815–1825

*© Cancer Research Campaign 1999*

Schalling M, Ekman M, Kaaya EE, Linde A and Biberfeld P (1995) A role for a new herpes virus (KSHV) in different forms of Kaposi's sarcoma. *Nature Med* **1**: 707–708

Shibata D and Weiss LM (1992) Epstein–Barr virus-associated gastric adenocarcinoma. *Am J Pathol* **140**: 769–774

Shibata D, Weiss LM, Hernandez AM, Nathwani BN, Bernstein L and Levine AM (1993) Epstein–Barr virus-associated non-Hodgkin's lymphoma in patients infected with the human immunodeficiency virus. *Blood* **81**: 2102–2109

Souza RF, Appel R, Yin J, Wang S, Smolinski KN, Abraham JM, Zou TT, Shi YQ, Lei J, Cottrell J, Cymes K, Biden K, Simms L, Leggett B, Lynch PM, Frazier M, Powell SM, Harpaz N, Sugimura H, Young J and Meltzer SJ (1996) Microsatellite instability in the insulin-like growth factor II receptor gene in gastrointestinal tumours. *Nature Genet* **14**: 255–257

Strickler JG, Zheng J, Shu Q, Burgart LJ, Alberts SR and Shibata D (1994) p53 mutations and microsatellite instability in sporadic gastric cancer: when guardians fail. *Cancer Res* **54**: 4750–4755

Tokunaga M, Uemura Y, Tokudome T, Ishidate T, Masuda H, Okazaki E, Kaneko K, Naoe S, Ito M, Okamura A, Shimada A, Sato E and Land CE (1993) Epstein–Barr virus related gastric cancer in Japan: a molecular patho-epidemiological study. *Acta Pathol Jpn* **43**: 574–581

Watanabe H, Enjoji M and Imai T (1976) Gastric carcinoma with lymphoid stroma. Its morphologic characteristics and prognostic correlations. *Cancer* **38**: 232–243

Wojtowicz Praga S (1997) Reversal of tumor-induced immunosuppression: a new approach to cancer therapy. *J Immunother* **20**: 165–177

Yamamoto H, Sawai H and Perucho M (1997) Frameshift somatic mutations in gastrointestinal cancer of the microsatellite mutator phenotype. *Cancer Res* **57**: 4420–4426

Yuen ST, Chung LP, Leung SY, Luk IS, Chan SY and Ho J (1994) In situ detection of Epstein–Barr virus in gastric and colorectal adenocarcinomas. *Am J Surg Pathol* **18**: 1158–1163

*© Cancer Research Campaign 1999*
